# Lord Tony Sewell

**DOI:** 10.1192/bjb.2024.72

**Published:** 2024-12

**Authors:** Abdi Sanati

**Figure:**
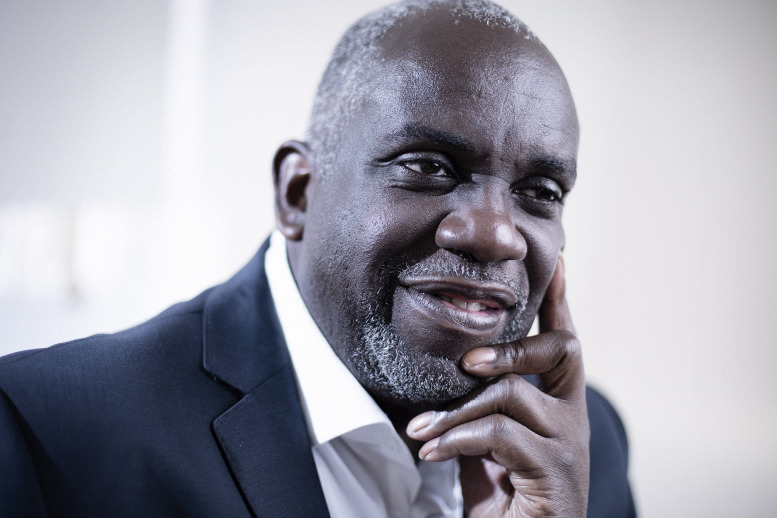
Credit: Rii Schroer.

Lord Tony Sewell was chair of the Commission on Race and Ethnic Disparities. He is also an international education consultant and chair of Generating Genius, which is a charity helping young people from disadvantaged backgrounds to excel in science, technology, engineering and mathematics (STEM) careers. I recently read his book *Black Success: The Surprising Truth*,^1^ and I found it interesting and engaging. I managed to have a conversation with Lord Sewell, thanks to technology, when he was in Jamaica.

**Dr Sanati: Many thanks for the interview, Lord Sewell. What I found very interesting in your book is the positive picture you have given for life of ethnic minority migrants in Britain. It was more or less against the mainstream picture of presenting life of ethnic minorities in a negative way. What motivated you in presenting this picture?**

Lord Sewell: In my life in Britain, I, and people I knew, were not defined necessarily by racism or, in fact, by race. There were so many other things going on in our lives that were positive. And it seemed to me that that story is just not being told at all. There were three things that pushed me towards that. Number one was the fact that in 1981 when I finished university, the country was different from now. It has significantly improved. There was overt racism at that time, and opportunities are better now for ethnic minorities. And the second thing was that I wonder whether we had not taken advantage of the real opportunities that were there. Race can become a comfort blanket for people, and they stay in that space. And they are frightened, because of the narrative, to go out and explore and do other things. I think there's a kind of impediment that comes along with race. The third thing I want to say is that the collective identity became linked to victimhood. So that Blackness itself, instead of what it was originally, part identity, part culture, became a kind of mass victimhood. So even when people were talking about their achievements, they felt they had to talk about achievement despite race. They couldn't think of their achievement as just their own agency. Those things were falsehoods. And I said to myself, what I want to do now is write something which will tell the truth.

**Dr Sanati: In recent years, we have witnessed the rise of the narrative of victimhood in society. In my own profession it was mirrored by the rise of the concept of trauma and its dominance. Do you think there is a pressure for migrants to take the victim role?**

Lord Sewell: It is complicated. Recent migrants only see opportunity and are prepared to sacrifice. They may share the same skin colour as more indigenous groups, but they are not invested in or aware of deeper struggles. Here is the irony, and it is a big irony, that the more entrenched the group is inside the society, the more propensity they have to victimhood.

**Dr Sanati: What I particularly liked about the book was the emphasis on agency. I have to say that as a migrant I never felt without agency. However, there is a negative determinism that dominates the narrative around migrants. Why do you think this is the case?**

Lord Sewell: I think what we've got in Britain are certain people, individuals and activist groups, who have grabbed the systemic racism narrative, mainly from the USA. Even though they are beneficiaries of the society themselves, and doing very well, they will tell you that Britain is so racist that it is almost impossible to use your own agency to do well. There's also another thing – the universities, the teachers and even artists are driven by social constructivism. And in that social constructivism you just look at the society like a prison. It is mostly based on Foucault and Marx among others, where you just look at the whole society as one big deterministic kind of machinery. Race is the key one because it is driven by White privilege. Power is determined by Whiteness, and Black and Brown people will never be free unless they sever those power relations. This is simply wrong.

**Dr Sanati: Another issue that caught my attention in your book was the importance you gave to the family as an institution. I have to say growing up in war and revolution, one thing that helped me as a child and then as a teenager was my family. Unfortunately, family has been ignored in the political discourse. How did we get to this point?**

Lord Sewell: So, in the 1970s and 1980s we had a narrative that said all our troubles were to do with single parents, single mothers in particular. We later felt a sense of collective guilt. So, what happens then is that society moves on, and we then go to the other extreme. We are now frightened to engage the family in terms of looking at some of the reasons why, for instance, you have poor educational outcomes. When it comes to exclusions in schools and children's different outcomes, you have to look at the family. Because we made the single parent the enemy number one at first, they now cannot be touched. And yet, I think we did a disservice to the children. This led to a dangerous liberal view in education and psychological research, where we wrongly thought that looking at the family was blaming the victim.

My mother was the major driver in my own life. So, what I'm hoping to restore is the notion of the family. Not to give the single mother a kick, but to understand and support families that are in crisis. When it comes to the Black experience in Britain, we have wasted a lot of money on programmes and interventions and yet we haven't had any change in outcomes. Many of those interventions have been mainly to do with trying to tackle racism. What we did not do was look at peer group, family formation, cultural influence and basic motivational skills. And so, a whole generation of young men have gone to prisons and mental institutions because we didn't bother to intervene in terms of the humanity of young people. This is the reason it's vital to look at Black success outside of notions of race.

**Dr Sanati: Your expertise is in education. What do you think of the calls to decolonise different curricula?**

Lord Sewell: Terrible. What is happening here is there is a White elite, and a certain Black middle class, who feel they have to get on this project, to cleanse the curriculum or the reading list and bring in something else. The issue for me is that you cannot solve a problem with something which isn't a tool to solve it. So, for example, if the problem you're trying to solve is working class or Black children not engaging in the curriculum in schools, then you're not going to solve it by banning Shakespeare. This doesn't make any sense because all you are doing is taking away knowledge from the children.

Then they argue that the curriculum is too White, to orientate it to a certain group, and they are trying to make it more relevant for those groups. But there lie lots of problems with that analysis. You are only indoctrinating your children or giving them your particular perspective when they need to know about everything. So, to take that away is almost to deprive those children. If you don't agree with something, you should actually still have that in there, so that you can understand it. We need to decolonise the de-colonisers.

**Dr Sanati: As minorities, we have been called many names, BME [Black and minority ethnic], BAME [Black, Asian and minority ethnic], among others. The final one which I really detest is the term ‘minoritised’. It totally takes the agency away from the person. What do you think of this term, and can you recommend a better term?**

Lord Sewell: Sounds like ‘monetarised’! These terms are going to come up because at the end of the day, it is in the interest of some to have as many people as possible under that victimhood kind of blanket. It is more complex. Even the term ‘Black’, I would argue is not useful. Within that you have different groups like Caribbean, mixed heritage and African, with different experiences and heritage. I find it quite interesting because those people who use it also talk about intersectionality. On the one hand, you talk about all these intersections, the next minute, you're using these big blanket terms, so which one is it? I think it's ideology of a political elite that sees Black and Brown and anybody not White all in one big camp, and fighting against the White world.

**Dr Sanati: Immanuel Kant once said human beings should be treated as an end in themselves and not as a means to something else.^2^ In your book you used the term ‘race hustlers’, who, if I read it correctly, use minorities as a means to their ends. How can we reclaim the status of being an end in ourselves?**

Lord Sewell: The answer is to do with narratives and stories that you tell about yourself, that you tell your children and that you share in schools. That's why I love Shakespeare. That's why I love Chaucer. That's why I like those Biblical stories, because all of them are about agency and how people have used their self-affirmation and resilience and have tested themselves against the odds and come out winners. We need to retell the stories that are not just mythological but also grounded within our own experience. And that's why I use the example of that Windrush generation. Ironically when I was writing, it was in the middle of the Windrush scandal. And it was a terrible issue, and they should have had their compensation. And what Theresa May did was wrong. What happened is that unfortunately the whole Windrush story got completely captured by that narrative of victimhood. So, you didn't hear about the resilience of the group that came over or how they managed to buy their own homes or how they did so well in face of a lot of hostility. So, instead you need to keep telling the narrative to yourself and to other people that you do have the ability. The other question is, what is stopping everybody from moving forward? I think when you are so used to being locked in this narrative of victimhood and being mentally imprisoned, you end up quite frightened, when in fact, the society is now much more open. How do you actually then exist? And one of the things I've noticed is the amount of Black people who then go for jobs that are in the Diversity and Inclusion industry. Even in places like big investment banks or in tech companies they go for jobs like Chief Inclusion Officer. What you need to do is take on the job where you are in charge of the assets in the company, because that's where things are progressing. But because you are linked to this whole narrative of victimhood you don't progress. You need to be able to have the courage and the confidence to grab the new freedom and not look back.

**Dr Sanati: You grew up in London and have been working with youth for years. In London, Black people are much more likely to be detained under the Mental Health Act. They are also more likely to be secluded or end in forensic pathways. What is your opinion on this and what do you suggest for improving this situation?**

Lord Sewell: The problem is what I mentioned earlier. When it comes to them, we are all too late. We get to them in the moment of crisis. We do not intervene early enough. When I taught in secondary schools, I could see children who would end up in the mental health system. One of the reasons is that we did not have interventions in the early period when we could see indications coming through. So, for example, you can see a link between the exclusions in school and those children who end up being sectioned. The seeds of trauma could have been spotted in school. Why didn't we have interventions that were not about wasting our time trying to do unconscious bias training with teachers, and really having interventions in the family? I am absolutely convinced that if you check the family history of those Black people who are currently in the mental health system, the trauma had a root in the family root rather than in a racist school. The second reason is more subtle. And I think it's to do with the fact that there is a large stigma attached to using those services. What happens is that you have a lot of young men who have no social or psychological support to help them when they have trauma. I did a little survey of who uses therapeutic services. Relatively more women use it than men, but hardly any Black males use those kinds of services. There's a stigma around those services. I think it is wrong for activists to suddenly turn around and say that the disparities are only due to systemic racism, when in fact, it's much more complex.

**Dr Sanati: Your report on race and ethnic disparities generated negative responses from many quarters. If you did care to respond to them, what would you say?**

Lord Sewell: I will tell them please read the report because they didn't read it. Because if they'd read it they would see that we never denied the existence of institutional racism. So, where they got that information from, I don't know. But I'd like you to quote that first sentence in that response of the College, where they say that we denied the existence or suggestion of institutional racism. We didn't mention it. And in fact, if anything, we imply that structural racism does exist. Otherwise, why give 15 recommendations including the Office for Health Disparities? I mean, if you're denying the existence of structural racism, why create an agency called the Office for Health Disparities? I think they wanted to connect the UK with the US protests. They didn't have the courage to say that our situation was different. The report followed the data and made a distinction between disparities and discrimination. In fact, quite clearly there was an issue to do with racism in the mental health system; however, things are much more complex than that. We saw health issues linked to an array of variables particularly people's geography and income. We looked across the range of things as well as race. So, I just think that particularly that College's response to our report was, to some degree, mischievous, and false.

Dr Sanati: Let's end on a humorous note. In *Les Misérables*, Victor Hugo quotes General Cambrone shouting ‘Merde’ at the British at the Battle of Waterloo. Milan Kundera writes, in *The Unbearable Lightness of Being*, that theology's biggest problem is not evil, but s**t. Fela Kuti wrote the song ‘Expensive S**t’ to criticise Nigerian police. You also commented on excremental matter in your book. Why does it have such rhetorical power?

Lord Sewell: Let's call it fertiliser! The idea is that it doesn't deny the experiences you're having, especially the bad ones. But the fertiliser itself can actually then be the source of something new and it's back to agency. I saw my parents do this. In the 1950s when my parents came over here, there were these signs outside of people's houses that said, ‘no Blacks, no Irish, no dogs’. They legally could put that outside a window. So basically, Black people couldn't rent. So, they were forced to go on to the housing market and buy properties. They saved up together and bought those houses. They became eventually the biggest owner-occupiers of all the ethnic minority groups. And even further down the line, when housing prices increased, they cashed in and did very well. And so here is an example of the negative being turned into the positive. I'm not justifying the existence of that kind of racism or saying that they should have experienced that. However, they used their agency and their will. They used it as an opportunity. So, you are right, s**t happens, but it's how you use it.
